# Borderline personality: revisiting its classification as a neurodevelopmental disorder

**DOI:** 10.3389/fpsyt.2025.1587778

**Published:** 2025-06-26

**Authors:** Olivier Laurini, Perrine Strugarek, Hassan Rahioui

**Affiliations:** Service des Troubles du Neuro-Développement chez l’Adulte (STNDA), Groupe Hospitalier Universitaire (GHU) Paris Psychiatrie et Neurosciences, Paris, France

**Keywords:** borderline personality disorder, neurodevelopment, ethiopathogenesis, childhood, adolescence, developmental, diagnostic criteria

## Abstract

Although borderline personality disorder is classified as a personality disorder, many studies have developed arguments in favor of a neurodevelopmental origin. In order to methodologically advance this new conceptualization of the disorder, we have identified six criteria for inclusion in the diagnostic category of neurodevelopmental disorders and propose to examine the extent to which borderline disorder may meet these criteria, illustrating them with recent studies.

Our review allows us to put forward the idea that this pathology could be considered as a late-onset neurodevelopmental disorder, present in childhood but fully manifesting in adolescence, a critical and rather explosive period of the developing brain. Such a perspective could help with early detection and treatment.

## 1 Introduction

Borderline personality disorder (BPD) is characterized by intense emotional instability, marked impulsivity, fear of abandonment and feelings of emptiness (1). This disorder is rather prevalent: it affects 0.7 to 2.7% of the people in community regional samples (2–4), between 10 and 12% of psychiatric outpatients and 20 to 22% of psychiatric inpatients (5). It is currently included in the category of personality disorders in the major international classifications.

Research has long focused on describing and understanding symptoms in adult populations. Still, in the last two decades or so, there has been a growing interest in understanding the etiology and early mechanisms of BPD.

In parallel, a conceptual evolution in personality disorders seemed to be underway through the exploration of a neurodevelopmental origin (6, 7). According to this hypothesis, the disorder results from objective abnormalities in the development of the central nervous system (growth, differentiation, lesions) and is influenced by genetic and environmental factors. Accordingly, it has already been proposed to reconsider antisocial personality disorder as a distinct neurodevelopmental disorder (NDD) (8). Could the same be true for BPD? Several literature reviews have already discussed the neurodevelopmental dimension of BPD (9–12), but to our knowledge they have not looked for operational inclusion criteria.

The purpose of this article is to conduct a literature review in order to answer the following questions:

Are there any existing inclusion criteria for NDD?

To which extent can BPD meet these criteria?

What study findings are available on children and adolescents?

This review will focus on articles providing recent data on risk factors, imaging, biology, genetics and neuropsychology.

## 2 Method and results

To conduct this review, we mainly used the PubMed server, searching for all articles published before July 2024 but prioritizing publications produced after 2010. The following syntax was used (borderline personality disorder) AND (developmental) OR (neurodevelopment) OR (neurodevelopmental). We selected the most relevant articles (meta-analyses, control group studies, systematic reviews) and also used the references of the articles found to complete the search.

We also extracted keywords from the articles (amygdala, hippocampus, imaging, childhood, adolescence…) and searched the database again as follows: (borderline personality disorder) and (amygdala) OR (hippocampus) OR (imaging) OR (childhood) OR….

We also looked to see if the question had been addressed with other personality disorders: (personality disorder) AND (developmental) OR (neurodevelopmental) OR (neurodevelopment).

Following a demonstrative approach, we will first expose a conceptual elaboration of the definition of NDDs and then discuss whether BPD meets these diagnostic criteria.

### 2.1 Definition of NDDs

Brain development, also known as neurodevelopment, refers to all the mechanisms of growth, differentiation and interconnection of the structures of the central nervous system. It begins in the embryonic stage and continues into early adulthood. It is a dynamic process based on neuroplasticity and influenced by biological, genetic and environmental factors (13–15).

Brain plasticity, or malleability, is the capacity for structural and functional evolution of the central nervous system. This process is found in both healthy and diseased circumstances. It can take place at different levels - anatomical, cellular (synaptogenesis, dendritic and axonal growth) or molecular - and according to different etiological mechanisms, sometimes combined (genetic, physical or chemical lesion, adaptive psychological, etc.). It varies across developmental stages, defining critical periods (16, 17).

The idea of linking a psychiatric condition to a problem of brain development is reported to have appeared in France in 1820 in the person of the alienist doctor Etienne Georget, a disciple of Philippe Pinel (18). The term “neurodevelopmental disorder” entered scientific usage in the late 1960s (19).

The term was first used in the third edition of the Diagnostic and Statistical Manual of Mental Disorders (DSM) in 1980 in relation to autism (20). It became a diagnostic category in the fifth edition (21). Some of the disorders were previously described in the DSM-IV-TR (22) as “Disorders usually first diagnosed in infancy, childhood, or adolescence”. In the ICD-10 (23) we found the title: “behavioral and emotional disorders with onset usually occurring in childhood and adolescence”. In a 2017 report, the English organization NICE (24) defined NDD as: A group of problems that become apparent during child development and often occur together. They are characterized by impairments of personal, social, academic or occupational functioning, ranging from very specific limitations to global impairments of social skills or cognition, as measured by parent or teacher reports and surveillance tools”.

According to the DSM-5-TR, NDDs are “a group of conditions with onset in the developmental period. The disorders typically manifest early in development, often before the child enters school, and are characterized by developmental deficits or differences in brain processes that produce impairments of personal, social, academic, or occupational functioning. The range of developmental deficits or differences varies from very specific limitations of learning or control of executive functions to global impairments of social skills or intellectual ability” (1). A number of criteria can be extracted from this definition: age, abnormality of brain development, functional impairment and neurocognitive impairment. There is no etiological criterion.

The ICD-11 (25) gives a rather similar definition: “behavioral and cognitive disorders that arise during the developmental period that involve significant difficulties in the acquisition and execution of specific intellectual, motor, language, or social functions”.

The most comprehensive and systematic definition was found in an article on the neurodevelopmental nature of antisocial personality disorder (8). This definition draws on scientific publications (21, 26–28) to propose a list of six inclusion criteria.

“A neurodevelopmental disorder

1. has its origins in childhood, frequently before grade school;
2. is characterized by abnormalities in brain structure and function throughout development;
3. is accompanied by neurocognitive impairments;
4. has a significant genetic basis;
5. runs a relatively stable course throughout development, without remission or relapse; and
6. continues into adult life, resulting in impaired social, academic, or occupational functioning.”

This list includes all the criteria present in the DSM-5-TR definition. In addition, within the temporal criterion, it distinguishes between age of onset and progression. It also includes a genetic origin, but does not explicitly mention environmental interactions. However, a number of publications on NDD emphasize these interactions (29, 30). We therefore propose to use Raine’s criteria to characterize NDD, with the exception that we will extend criterion 4 to include gene-environment interactions.

After identifying these six features, let us successively examine whether BPD satisfies them.

### 2.2 Criterion 1: Is the disorder present since childhood?

The conceptualization of BPD as NDD implies considering its presence since childhood.

#### 2.2.1 Personality disorders and childhood

Personality disorders are a category generally reserved for adults. However, ICD-10 (23) states that personality disorders “usually manifest since childhood or adolescence and continuing throughout adulthood”.

The third edition of the DSM (20) states that personality disorders, by definition, begin in childhood or adolescence and does not prohibit diagnosis before the age of 18 as long as the traits are persistent, while noting that the stability of the diagnosis is uncertain. The DSM-IV (31) specifies the need for a duration of development of at least one year and a pervasive nature.

In routine clinical practice, it is not uncommon to seek such a diagnosis in relatively young people, as evidenced by the existence of adapted and validated scales for detecting personality disorders in children (32).

In terms of BPD itself, the DSM-5-TR no longer considers it to be an entirely adult-onset disorder: it reports that in clinical practice, pre-adolescents aged 12–13 years meet all the criteria (1).

The diagnosis of BPD in adolescents has long been controversial, but is now well established (33, 34). In children, the validity of this diagnosis is still debated for two reasons: from a clinical point of view, the personality is considered to be developing, and from an ethical point of view, a stigmatizing label would be placed on the child (35).

#### 2.2.2 Adult and child: is it the same disorder?

The definition of BPD can vary between authors and schools of thought. The term borderline appeared in 1938, applied to adults (36), to describe a borderline state between neurosis and psychosis. A clear distinction between BPD and psychosis (“borderline schizophrenia”) has only recently been established, with detailed criteria (37). BPD appears in the third edition of the DSM in the section on personality disorders (20).

The first clinical case description of BPD in children dates back to the 1940s with the case of Velia (38). The term borderline child was first used in 1954 (39).

The syndrome of borderline children has features in common with psychosis. The difficulty in understanding and classifying this disorder seems to argue for a neurodevelopmental dimension. Thus, in the 1980s and 1990s, the inclusion of childhood borderline disorder among personality disorders was debated in the name of organicity (40, 41). For a time, it was included in the group of MCDDs (Multiple Complex Developmental Disorders). These disorders were defined by three criteria: impaired emotional regulation, impaired social interactions with hypersensitivity, and impaired cognitive processes (42, 43). This category has subsequently fallen into disuse (44).

Researchers have long questioned whether what is described in children and adults really represents continuity of the same disorder (40). A comprehensive review of the literature comparing BPD in children and adults was undertaken in 2005 (45). It concluded that the similarities outweigh the differences. Similarly, a 2016 meta-analysis (46) concluded in favor of the validity of the diagnosis of BPD in children and adolescents (under 19 years of age) due to shared etiological and pathophysiological features.

#### 2.2.3 Diagnostic criteria

Assuming that it is the same disorder, an initial problem was the appropriateness for children of diagnostic criteria originally designed for adults (47).

The scales were adapted with slightly reformulated DSM III criteria (48).

In 2003, Zanarini published a scale called the Children interview for DSM IV Borderline personality disorders (CI-BPD), which was subsequently tested and validated. It uses simplified language turns (49).

In children, this condition is more carefully referred to as borderline personality traits rather than a personality disorder. These traits can be measured with some reliability (50). This has led to the proposal of the concept of the *borderline-child-to-be* (35).

Crick et al. (51) developed the Borderline Personality Features Scale for Children (continuous) for children aged 9 and over, an adaptation of the Personality Inventory (PAI) scale (52). It consists of 24 items that give a score. It has been translated and validated in several languages (53).

#### 2.2.4 Age of onset

In one of the first descriptions of early BPD, the case of Velia, the child is 7 years old (38).

For Judd (54), it is possible that the disorder is an early-onset pathology that has been subtly present in the individual for a long time. It would only be recognized in childhood in its most severe forms, when it would become truly visible and invasive. It would emerge in its full and invasive form in adolescence, when social and developmental demands would exceed the subject’s abilities.

To our knowledge, few studies have attempted to estimate the average age of onset of BPD. The DSM-5-TR states that the average age of onset is not known (1). Some longitudinal studies suggest that it may begin in early adolescence (50, 55, 56).

Zanarini reports that 32.8% of people with BPD started self-harming before the age of 12, and 30.2% between the ages of 13 and 17 (57).

In any case, the disorder does not emerge *ex nihilo* in adolescence, and there are certainly warning signs and risk factors in children.

#### 2.2.5 Epidemiological studies

The main difficulty is the lack of data: our research found relatively few studies in children. Science is seemingly more focused on risk factors present in childhood than in the clinic (58).

When they exist, epidemiological studies are not always conducted in the general population, but in heterogeneous samples of hospitalized patients (59), in which children and adolescents can be mixed (46).

A prevalence study in a community population of 9–19 year olds found a figure of 3% (60).

A British cohort of 6,330 children followed since birth showed a prevalence of about 3% at age 11 years according to DSM-IV criteria (61).

In a community sample of Quebec patients aged 12 to 14, the observed prevalence is 4.2% at 12 years, 5.7% at 13 years and 9% at 14 years (62).

A Danish study conducted between 2007 and 2017 (63) showed that BPD is the most common personality disorder (between 23.6% and 34.1%) in people under 18 years of age receiving psychiatric treatment.

### 2.3 Criterion 2: Abnormalities of brain structure and function

By definition, NDDs require abnormalities in brain development. Are there structural and functional brain abnormalities in BPD? Are they present from early childhood?

To our knowledge, studies that focus solely on childhood are quite rare. Yet, we found one comprehensive systematic review (46) that focused on neurobiological research in children and adolescents.

#### 2.3.1 Abnormalities seen on imaging

Research and findings mainly concern the frontolimbic regions and circuits that are thought to be associated with BPD (64). A recent publication (65) lists the results of structural and functional MRI imaging in adolescents. It includes 14 studies whose populations were mainly drawn from a few cohorts (Orygen in Australia, Heidelberg University, Mount Sinai Hospital and Montreal Hospital). At best, imaging results are only available at the time of the first consultation, but the disorder may have been present for several years.

##### 2.3.1.1 Limbic system

The hippocampus, amygdala and cingulate gyrus are part of the limbic system, which is thought to be involved in emotional and behavioral responses (66).

In adults, structural imaging generally shows a reduction in the volume of the amygdala and hippocampus (67). However, not all studies are consistent (68).

Studies conducted in adolescents with BPD are fewer in number and smaller in sample size. Data are less reliable (69). No results were found for children.

In the ORYGEN cohort no reduction was found in amygdala or hippocampal volume compared to controls, suggesting that this reduction would occur as the disorder progresses. The study included two groups of 20 subjects aged between 15 and 19 years (70).

One study showed a significant volume difference in the hippocampus (bilateral) and the right amygdala (71) in girls aged 14 to 18 years.

The study by Jovev (72) on a sample of 153 children with a mean age of 12.6 years (minimum 11.4 years, maximum 13.7 years) reports an association between atypical hippocampal symmetry and impulsive behavior (BPD and antisocial personality).

##### 2.3.1.2 Cortical abnormalities

Abnormalities of the prefrontal cortex can lead to difficulties in recognizing emotions and modulating behavior through rational decision-making (66).

In adults, reductions in cortical volume are found in the anterior cingulate cortex, prefrontal cortex and orbitofrontal cortex (73). Again, the data are fragile (68).

In adolescents, one study showed a loss of grey matter in the right prefrontal cortex (70). Another study found significant bilateral shrinkage of the prefrontal cortex (74).

A decrease in the volume of the anterior cingulate cortex is found in adolescents with BPD (75, 76) or who exhibit self-aggressive behavior (77).

Two recent studies of borderline adolescents (78, 79) found systematic reductions in cortical thickness in the frontolimbic circuit and the default mode network (which includes the posterior cingulate, medial prefrontal cortex, and angular gyrus, among others). An inversion of right/left symmetry is observed in the orbitofrontal cortex.

##### 2.3.1.3 Abnormalities in functional reactivity

Functional MRI can be performed at rest (“resting state”) or during the performance of tasks (looking at pictures) designed to evoke an emotional response.

Studies in adults have shown an imbalance in the pattern of activation between frontal and limbic structures (80). Thus, the amygdala, the hippocampus and the anterior cingulate cortex show hyperreactivity (81, 82), whereas the frontal structures would be less reactive. Those results are controversial (81). One study highlighted a reduction in functional reactivity of the amygdala and anterior cingulate cortex following psychotherapy (83). In adolescents, a feasibility study was published in 2016 (84) and a study on functional reactivity was conducted in 2018 (85) with tasks inducing feelings of social rejection. Hyperactivations in the posterior part of the left insula and the left dorsal striatum as well as in the left inferior frontal cortex were observed in adolescent girls.

##### 2.3.1.4 Abnormalities in functional connectivity

Functional connectivity is an MRI technique that makes it possible to locate brain structures that function in a network. It is demonstrated by a statistical correlation between measurements of their activity.

Studies in adults show abnormalities in the default mode circuit, the salience network and the central executive network (86).

In adolescents, recent studies also show an alteration in functional connectivity (78, 79) of regions related to emotional regulation.

#### 2.3.2 Biological abnormalities

##### 2.3.2.1 Opioid system

Alteration of the endogenous opioid system is a hypothesis investigated in the pathophysiology of BPD. This disruption is reflected by an increase in the sensitivity of µ-opioid receptors in brain regions associated with emotion and behavior, which may be involved in BPD (87).

Symptoms of borderline disorder can be interpreted in terms of the opioid system (88). Seeking attention could be a way of activating the reward circuitry. Feelings of emptiness may reflect a reduction in the activity of the system. Substance abuse involves substances that target µ receptors. Finally, behavioral disorders may be ways of putting the body into survival mode to mobilize the reserves of the opioid system. A relatively small number of studies have looked at this. Treatment of borderline patients with opiates (buprenorphine/naloxone) has been considered, but has not yet been scientifically proven to be effective (89).

##### 2.3.2.2 Hypothalamic-pituitary-adrenal axis

At the neuroendocrine level, the HPA axis is involved in stress response mechanisms. According to the most widely accepted theory, early traumatic experiences induce chronic hyperactivation of the corticotropic axis, which could then lead to an alteration in its functioning in adolescence in the form of desensitization (burn-out effect).

Two reviews were published in 2019 on this topic, including a meta-analysis (90, 91). People with BPD were found to have elevated baseline cortisol levels and a blunted response to stressors. The results are not all consistent due to confounding factors and different ways of measuring corticotropic axis activity (pituitary volume, salivary cortisol, morning secretion peak, ACTH test, etc.). One study (92) examined pituitary volume in adolescents presenting for the first time with BPD (n=20, mean age 17.3 years), but found no difference from the control group (n=20). However, a difference was found within the BPD group for history of trauma (n=9).

In adolescent girls (14–18 years) prone to self-aggressive behavior, cortisol secretion in response to stress is lower than in the control group (93).

##### 2.3.2.3 Thyroid gland

Recent studies show no association between thyroid dysfunction and BPD (94, 95).

##### 2.3.2.4 Brain-derived neurotrophic factor

BDNF is a growth factor involved in neurogenesis, synaptogenesis and brain plasticity. Its role is often studied in neurological and psychiatric pathologies (96, 97).

BDNF levels (measured in platelets) have been found to be reduced in patients with BPD (98), suggesting impaired brain plasticity. An alteration in the habituation mechanism of the amygdala response could also be caused by BDNF deficiency (99).

##### 2.3.2.5 Oxytocin

Oxytocin is a peptide hormone produced by the hypothalamus. It plays a role in social cognition, behavior and emotional capacity. Its secretion is thought to reduce anxiety and modulate the stress response to social interactions (100).

Several studies have been conducted in patients with BPD. A 2022 meta-analysis (101) compared oxytocin levels in groups of patients with psychiatric pathology. A decrease in oxytocin levels is found in particular in BPD patients and in patients with schizophrenia, while increased concentrations are observed in other conditions (bipolar disorder, obsessive-compulsive disorder).

These differences may be due to abnormalities in the signaling pathways of this neuropeptide. One gene-environment interaction has been studied: a link between childhood maltreatment and the OXTR gene, which is involved in the coding and activity of the oxytocin receptor (102).

A 2024 study (103) examined biological markers in adolescent patients (12 to 19 years old) with self-aggressive behavior and reported a decrease in oxytocin levels as an important factor.

Several studies have suggested a benefit of intranasal oxytocin treatment on emotion regulation in BPD (104, 105). These findings are not supported by other studies (106).

### 2.4 Criterion 3: Neurocognitive deficits

The presence of neurocognitive symptoms may reflect impaired brain function and suggest the presence of an underlying neurodevelopmental disorder. There are more studies using neuropsychological tests than biological studies in children with BPD (46). The most commonly affected neurocognitive domains are executive functions and social cognition (107).

#### 2.4.1 Executive functions

Executive functions include planning, decision-making, inhibitory control and mental flexibility (1).

In a neuropsychological study of children with BPD aged 7 to 12 years, abnormal scores on the Wisconsin Card Sorting Test and the Continuous Performance Test indicated impaired planning skills (40).

Zelkowitz confirmed these data using the same two tests in 2001, and concluded that understanding the disorder must take into account both environmental risks and neurobiological vulnerability (59).

In a cohort of children with borderline traits at age 12 who were assessed at age 5, the executive functions tested (planning, working memory and inhibitory control) are slightly lower (108).

#### 2.4.2 Social cognition

Impairment in social cognition can be demonstrated by deficits in emotion recognition and theory of mind.

BPD patients have been tested with the RMET (reading the mind in the eyes test), which involves recognizing and discriminating facial emotions, thoughts and feelings (107). Their performance is significantly lower than that of the control group.

Theory of mind (or mentalization ability) was tested in a cohort of 12-year-old children: it appears to be negatively correlated with the presence of borderline personality traits (108).

#### 2.4.3 Emotional dysregulation

Emotional dysregulation is central to BPD and has been the subject of numerous analyses (77, 109, 110). It consists of four factors: emotional hypersensitivity, tendency towards negative effects, poor understanding of emotions, and maladaptive management of emotions. It is central to Linehan’s theoretical model of BPD (111).

To negative emotions, borderline patients have an emotional response pattern that seems similar to post-traumatic stress disorder. The intensity of the emotions is greater and the return to baseline is slower. For positive emotion images, the pattern is closer to that seen in depression, the intensity being lower and the return to baseline quicker (112).

Rejection sensitivity is one of the key issues (113, 114). The cognitive components have been thoroughly studied (115–117). It may result from the combined effects of hypersensitivity and a deficit in social cognition leading to misinterpretation (118).

Coping and emotion management strategies appear deficient or inadequate: BPD patients make little use of cognitive reappraisal or acceptance, but are prone to repression and rumination (119).

### 2.5 Criterion 4: Genetic and environmental underpinnings

The combination of genetic and environmental factors is the most widely accepted theory for the etiology of BPD (9, 120).

#### 2.5.1 Heritability

A recent large Swedish population-based registry study (121) found heritability of 46% and increased risk in relatives. A three-country study found a heritability of 42% (122). In a cohort of 12-year-old twins, the heritability of borderline personality traits was quantified at approximately 66% (108).

Ruocco et al. (123) observed that siblings of BPD patients also showed symptoms at a subclinical level (emotional dysregulation, attentional impulsivity and cognitive instability). Thus, the presence of the disorder in different forms in different members of the same family supports the hereditary nature of the disorder.

#### 2.5.2 Genes involved

There is currently no consensus on the identification of specific genes for borderline disorder. In addition, all of the genes considered have been studied in isolation, preventing an overview of the possible dynamic interactions between the different causes (124). The most studied loci are polymorphisms on genes involved in the dopaminergic, noradrenergic and above all serotonergic system, particularly the short allele of the 5-HTTLPR gene (125). All of them are involved in impulsivity, introversion and negative affectivity (124).

#### 2.5.3 Epigenetics and the environment

##### 2.5.3.1 Epigenetics

DNA methylation at the BDNF gene appears to play a role in BPD. The methylation rate has been found to decrease following psychotherapy (126).

##### 2.5.3.2 Perinatal factors

A case-control study found an association between borderline disorder and pregnancy-related factors (127): exposure to tobacco, exposure to stressors, and obstetric complications. There are also perinatal risk factors for other psychiatric disorders, such as schizophrenia. The link is difficult to establish. A cohort study (128) highlighted anxiety and depression during pregnancy as risk factors for borderline disorders. After adjustment, alcohol and tobacco are not among the risk factors.

##### 2.5.3.3 Environmental factors

Individuals with BPD are thirteen times more likely to report child abuse than controls (129). A very comprehensive study focused on the risk factors for early-onset borderline disorder (130). According to this study, the four factors highlighted are:

-Sexual abuse

Many studies have established that children and adolescents who are victims of sexual violence are at greater risk of developing BPD (48, 131).

-Physical and verbal abuse

A cohort of twins tested the hypothesis that physical and verbal abuse before the age of 10 is a risk factor for the development of borderline personality traits at the age of 12 (108). However, not all studies show the same degree of correlation: according to Stepp (132), the correlation should also be considered according to the developmental period during which the abuse was suffered.

-Neglect

Neglect is the inadequate response to a child’s physical and emotional needs (demand for care and attention). BPD patients report high rates of prolonged separation from parents (or caregivers) (133), especially their mothers (134). They also report emotional neglect (135, 135). Liotti and Pasquini (136) found that the risk of developing BPD was 2.5 times higher in individuals whose mothers had suffered a loss within two years of their birth.

-Bullying

Bullying refers to physical and verbal violence perpetrated by peers. Early-onset BPD was found to be associated with bullying in a prospective longitudinal study (137, 137).

##### 2.5.3.4 Attachment disorders

Attachment disorders can reasonably be included among environmental factors. Indeed, they are directly related to the interactions between the child and his or her caregivers, who are most often part of the family environment. This assumption is supported by Hughes’ Social Baseline Theory (64), which emphasizes the importance of the interpersonal process in the development of BPD. Some kind of connection can be noticed between personality disorders and attachment disorders, particularly in the area of relationship dynamics. However, it is not possible to equate borderline personality and an attachment disorder (138).

For the record, in children, attachment is mainly assessed at the age of 18 months through Mary Ainsworth’s Strange Situation. Four child profiles are usually distinguished: avoidant (A), secure (B), ambivalent (C) and disorganized (D) (139).

Adult classifications are diverse and complex (140). On the one hand, one can find a two-dimensional (anxiety and avoidance) system based on questionnaires, leading to four profiles (Secure, Dismissing-avoidant, Preoccupied, Anxious-avoidant) (141). On the other hand, there exists a categorical system (Berkeley), based on the Adult Attachment Interview (AAI), which incorporates some of Ainsworth’s categories (ABC+D), but also comprises several other categories, including Unresolved and Cannot Classify (142).

According to the internal working models theory, attachment disorders can occur through an altered image of self and others: anxious attachment due to fear of abandonment, avoidant attachment due to lack of trust (143, 144).

Longitudinal studies have examined the relationship between infant attachment profiles and later borderline symptoms. Attachment style does not appear to predict borderline disorder (145). The isolated factor is parental behavior rather than attachment style (146).

Concerning attachment profiles measured in adults, a number of researchers have investigated the association with the incidence of BPD (147–153). For example, Barone examined attachment status in a sample of 80 individuals (40 BPD patients, 40 controls). He identified only 7% of people with BPD as autonomous, while 23% were preoccupied, 20% were avoidant and 50% were unresolved. This highlighted the similarities between BPD and disorganized attachment (154).

A review of the relationships between BPD and attachment disorders (138) identifies neurodevelopmental correlates that are shared by BPD and disorganized attachment. Functional imaging research has indicated that the limbic brain is also activated during tasks that stimulate the attachment system (155).

### 2.6 Criterion 5: Stability across development without regression or remission

To our knowledge, there are no very long-term longitudinal studies of the development of BPD from childhood to old age. Existing studies are not homogenous, and may involve different samples (clinical or non-clinical) or use different diagnostic scales.

As a personality disorder, BPD used to be considered chronic and not significantly modifiable. It is now accepted that the disorder generally improves with age (156).

The stability of the diagnosis between childhood and early adulthood (between 2 and 20 years) was examined in a systematic review (157). Of the ten studies reviewed, this stability is considered to be low to moderate. It ranges from 14 to 40%.

The condition apparently evolves during adolescence. In a population of young girls followed from 14 to 19 years of age (158), the disorder peaks at around 15 years of age, then decreases between 15 and 18 years of age and stabilizes between 18 and 19 years of age.

Subsequently, borderline personality traits have been found to decline with the transition from adolescence to adulthood, most notably in Bornovalova’s study (159), which examined the stability of diagnosis in a cohort of twins followed for 10 years (from age 14 to 24). In another study of adolescents, the diagnosis is still present in only 24% of cases after 5 years of follow-up (160).

In adults, the McLean cohort, which began in 1992, is the longest running study. The mean age at enrolment was 26.9 years (18 to 35 years). The authors distinguish between symptomatic remission and recovery, which is associated with a high Global Assessment of Functioning score. After 20 years of follow-up, only 39% of patients had an excellent recovery (161).

The expression of the disorders could vary according to age (55),with more behavioral symptoms and self-aggressive gestures in younger people (162, 163). Of the symptoms, chronic emptiness is the one that decreases the slowest (164) and has the lowest remission rate. Should we then assume that the disorder has completely disappeared if there are residual subsyndromal signs and global functioning is still impaired?

### 2.7 Criterion 6: Impaired social, academic and occupational functioning

This criterion overlaps with the general diagnostic criteria for personality disorders. As reported by DSM (in all its editions since DSM IV), the diagnosis of a personality disorder requires “a clinically significant distress or impairment in social, occupational or others areas of functioning” (1).

#### 2.7.1 Childhood and adolescence

The need for improvement in psychosocial functioning is frequently stated as rationale for early intervention in youth with BPD (165).

In a 2015 meta-analysis (157) addressing the “clinical and psychosocial outcome of borderline personality disorder in childhood and adolescence”, very few studies were reported dealing with psychosocial issues in childhood. One of them (166) investigated the 5 year outcome of borderline pathology of childhood (diagnosed with retrospective interview). Significant indicators of impaired functional status included the need to change schools due to behavior problems, out-of-home placement and problems with peers.

However, a number of research highlight the burden of BPD during adolescence in terms of quality of life and cost (167).

Chanen and al (168). compared adaptive functioning in three groups of adolescents aged 15 to 18 (BPD, other personality disorders and no personality disorder). The study used questionnaires such as the Social and Occupational Functioning Assessment Scale. The BPD group revealed the highest functioning impairment in many areas (peer relationship, medical problems, financial status and social support).

A 2016 publication (169) based on a community sample of girls between 14 and 17 highlighted an association between BPD symptoms and poor outcomes in academic performance, activity involvement and global assessment of functioning (home, school, with friends and during leisure time).

Many studies conclude that early onset BPD predicts poor functioning. A longitudinal cohort study (170) showed that children who met the criteria for BPD at the age of 12 were more likely to experience functional difficulties at the age of 18. Such difficulties include low life satisfaction, social isolation, low educational qualification and high official crime record.

A Danish register-based study (171) found that at the age of 20, early onset BPD adolescents were significantly more likely than controls be unemployed or to obtain a lesser level of education. The related health cost was also higher.

#### 2.7.2 Subsequent functioning impairment

Similar outcomes persist into adulthood, as reported by Winograd et al. (172): as compared to the general population, the academic trajectory of people with BPD is more chaotic and they do not achieve the same level of education. Few of them can pursue a leisure activity in the long term (173). A 10-year longitudinal study in Spain showed stability in functional limitations in participation in social life (174). People with BPD have higher than average unemployment rates (175, 176). Poverty rates are higher and people are more likely to be homeless (177). According to an American longitudinal study, only 25% are in full-time employment (178) and 40% receive disability benefits. Programs are being set up to encourage young people to return to work (179). When in work, people with BPD are more prone to absenteeism and perform less well on the days they are present (180).

They are more prone to suffer from exclusion and relationship dysfunction. Their social integration is less successful and their relational network less dense than the rest of the population (181). Relationship conflicts are more frequent. Only 16% live in a couple (182). They are more likely to be involved in legal disputes than the rest of the population (183).

Quality of life is below average (184). Scores on scales of functioning and social adjustment remain low even after symptomatic remission (178). Life expectancy is approximately 20 years less than the general population, due to suicide attempts and poorer physical health, including the presence of cardiovascular disease and eating disorders (185).

## 3 Discussion

The aim of this review was to take stock of recent findings that support the thesis that BPD can be defined as an NDD. Many elements support the hypothesis that borderline disorder is not a personality disorder that emerges in adulthood. Rather, it should be understood as a disorder that is present from an early age but whose symptoms explode in adolescence.

The number of criteria needed to confirm the diagnosis has not been defined. Studies do not currently allow us to conclude that the disorder appears at pre-school age (criterion 1). There are brain (criterion 2) and neurocognitive (criterion 3) abnormalities. There is a genetic and environmental basis (criterion 4). However, the disorder is not stable over time. The degree of stability can reach 40% (criterion 5) as with other psychiatric disorders. Social and occupational difficulties exist (criterion 6).

The criteria are therefore met, except for 1 and 5, which are only partially met. However, these two criteria should be re-examined regularly in the future as the means of investigation improve. Criterion 1 could be addressed in a particular investigation relative to the disorder’s age of onset. As for criterion 5, it requires a consensus definition of the stability over time, encompassing global functioning, residual symptoms and the impact of treatment.

### 3.1 A late-onset NDD?

It has been common practice to consider only those disorders that occur during embryonic life as developmental disorders (186). However, there is no specific age in the definition of NDD (“typically before school age” (1)). In DSM IV TR, autistic disorder required the presence of symptoms before the age of 3. DSM 5 does not have such a restriction regarding autism spectrum disorders (ASD). Criteria only point out “the early developmental period”. The “Development and Course” section of ASD reports that “the symptomes are typically recognized during the second year of life”, but may be seen earlier or later. Other NDDs do not always include precise age criteria. A definition provided by the US Social Security administratively sets a maximum age of 22 years (187).

The temporal boundaries of brain development are quite broad and do not overlap with those of childhood. Brain volume reaches its maximum in early adolescence. Adjustments continue during adolescence and beyond the age of 20 (188). In particular, the prefrontal cortex, which is involved in emotion management, is reported to be the last to mature.

BPD may therefore be a late-onset neurodevelopmental disorder.

### 3.2 Towards a stress-related model of development?

Much has been written about the consequences of childhood trauma and, in particular, its influence on early-onset borderline disorders (58). The most coherent synthesis, taking into account the largest number of all these clinical abnormalities, leads to a neurodevelopmental model of stress-related borderline disorder (91, 189).

Chronic stress associated with early trauma leads to overstimulation of the HPA axis and alteration of the harmonious developmental process and connectivity of frontolimbic structures. The consequences are all the more severe as trauma occurs during critical periods of neurodevelopment when the brain is particularly sensitive to external stimuli (190). A disruption of the hippocampus can lead to an erroneous and excessive interpretation of small stimuli as constant threats in the environment and cause hyperactivation of the amygdala. The latter plunges the organism into a disproportionate, unstable and unpredictable emotional state (191). At the same time, overstimulation of the prefrontal cortex leads to cognitive and executive dysfunction, resulting in impulsive and inappropriate behavior. This chronic stress also induces epigenetic changes.

Based on these findings, a stress-related mouse neurodevelopmental animal model of BPD was developed (192). The authors conceptualize BPD in terms of conditioning mechanisms, which implies an alteration of the processes of habituation to aversive stimuli and extinction of the fear response (193, 194). This is a two-step model: the authors note that a genetic background or early life stress is not always sufficient to explain the onset of the disorder; they postulate the presence of a so-called proximal stress, perhaps less intense, which would be present before the onset of the disorders.

The mice are first exposed to a wave of early stress (separation from the mother, etc.) between the first and 21st days of life. This is followed by a less intense stress in adulthood (after 60 days).

A psychological and behavioral assessment is then proposed, with specially adapted tests covering three areas: social interactions, emotional regulation and impulsivity.

This model is still in draft form and has not been tested.

### 3.3 How to include borderline cases without trauma?

The scientific community has long agreed that the experience of trauma in childhood, or at least an experience of major adversity, is a prerequisite for the development of borderline disorder. However, this view of the disorder has been challenged (54, 124, 195).

Abuse may not be a specificity of borderline disorder, nor a necessary or sufficient cause (196). In fact, 80% of individuals who have experienced sexual abuse do not meet the criteria for borderline disorder (40), and 20-40% of borderline individuals have never experienced neglect or abuse (195).

Studies highlight the fact that groups exposed to the traumatic risk factor develop the pathology in higher proportions than those who are not exposed. They also show that the unexposed also develop it (197). The authors emphasize the interconnectedness of risk factors and the difficulty of disentangling and isolating them. A significant proportion of the population is affected, and these nonconsensual trajectories remain an area of research that has yet to be fully explored empirically.

### 3.4 Associations with neurodevelopmental disorders

In recent years, there has been a growing interest in the literature to study the comorbidity of borderline disorders with neurodevelopmental disorders (54, 198, 199). Associations with ADHD and learning disorders are common. Comorbidity with autism spectrum disorders is also beginning to be studied (200).

As NDDs are often comorbid with each other, this association with certain NDDs raises questions about the nature of borderline disorders.

For ADHD, a literature review by Weiner et al. (199) addresses this issue. ADHD and BPD overlap on two key dimensions: impulsivity and emotional dysregulation, but also through impaired interpersonal relationships and low self-esteem. A significant proportion of ADHD patients (18 to 34% in adults) and borderline patients (16.1 to 38.1%) have both disorders.

This comorbidity seems too prevalent to be a simple coincidence. To explain this, the authors hypothesize common risk factors that are both genetic and environmental (201). A Swedish registry study has shown a familial association between the two disorders, suggesting a genetic dimension (202). It has been suggested that ADHD is a risk factor and an early form of BPD (203, 204). However, the boundaries between the two pathologies are now less clear than they used to be, and the results of different studies are not all consistent (205).

### 3.5 Therapeutic implications

Early intervention aims to increase the therapeutic effect and avoid chronicity. Identifying and taking into account the first symptoms as soon as they appear in childhood could therefore allow rapid intervention. It is particularly important to intervene at a moderate stage when the disorder is not yet fully developed. Limiting and minimizing the development of symptoms can be considered, without being hindered by the difficulties associated with the pathology, before it becomes more invasive. Supporting the progression of the disorder helps to promote a better entry into adolescence, a period when neurodevelopment continues through socialization with peers outside the family.

A recent review focused on the various options for early intervention (206). Prevention is divided into three branches: primary, secondary and tertiary. Primary prevention aims to limit the prevalence of the disease in the population. There are not enough interventions or studies to prove their effectiveness. Secondary prevention consists of intervention programs when the disease is not yet fully manifest. Tertiary prevention refers to interventions after the disease has been diagnosed.

Early intervention is promoted by organizations such as the Global Alliance for the Prevention and Early Intervention for Borderline Personality Disorder. The HYPE (Helping Young People Early) program has been implemented in Australia. A single-blind randomized controlled trial (207) compared three types of intervention for BPD (HYPE and specific therapy; HYPE and befriended therapy; and conventional management). All three types of therapy showed efficacy.

Two reviews examined interventions in adolescents (208, 209): their effects were found to be moderate (210).

However, a 2024 study (211) showed differential effects of early intervention in adolescence depending on age group, suggesting that intervention in subsyndromal forms could limit the onset of the disorder. Adapting therapy to age-related developmental specificities may optimize treatment.

### 3.6 Limitations

This work has several limitations. Many articles were consulted, but it is not a systematic review. Some of the references collected for this review are themselves literature reviews.

Research on BPD needs more empirical studies. The topic is still developing. The research collected shows results that are still scattered and partial. Too few studies specifically address childhood. There is a lack of longitudinal studies on the subject. It will be necessary to carry out studies on cases of patients without trauma. The populations studied are not homogeneous, they can be general or hospital populations. There are many confounding factors: treatments, comorbidities.

The lack of specificity of biological and anatomical markers is a problem. The risk factors are the same as for other psychiatric pathologies. For example, the processes involved in antisocial personality disorder are very similar. Studies of antisocial personality have more arguments, including the identification of the cavum septum pellicidum (8), which reveals an early abnormality in the development of the limbic system.

### 3.7 Conclusion

The arguments in favor of a neurodevelopmental model of the disorder can be methodically reviewed and conceptualized as six criteria to be discussed for an inclusion into NDDs. These criteria now need further research to be scientifically validated and adopted in common practice.

Both genetic and biological aspects, showing a certain heritability of the disorder, and environmental aspects, in particular difficulties in interactions with attachment figures, make it a disorder with multifactorial causes.

The study of the trajectories without trauma, the warning signs and the frequency of comorbidities in NDD support the idea of a disorder with an earlier onset than it appears and whose trajectory may differ from one individual to another.

According to our findings, the biological vulnerabilities of emotional regulation and impulsivity present from birth interact with the relational environment in which the individual would have difficulty adjusting, a fortiori when the attachment figures have the same problems. These emotional difficulties would increase as social demands increased during development. Compensation would become increasingly difficult, and the disorder would become fully manifest in early adulthood.

This neurodevelopmental perspective is crucial both for the stigmatization of the disorder and for its prevention, as it would facilitate early detection and treatment.
